# Immuno-metabolic impact of the multiple sclerosis patients’ sera on endothelial cells of the blood-brain barrier

**DOI:** 10.1186/s12974-020-01810-8

**Published:** 2020-05-09

**Authors:** M. H. Sheikh, S. M. Henson, R. A. Loiola, S. Mercurio, A. Colamatteo, G. T. Maniscalco, V. De Rosa, S. McArthur, E. Solito

**Affiliations:** 1 John Vane Science Centre, Barts and The London School of Medicine and Dentistry, William Harvey Research Institute, Queen Mary University of London, Charterhouse Square, London, EC1M6BQ UK; 2 Laboratoire de la Barrière Hémato-Encéphalique, Faculty Jean Perrin, EA 2465, Université d’Artois, Arras, France; 3 Dipartimento di Medicina Molecolare e Biotecnologie Mediche, Universitá degli Studi di Napoli “Federico II”, Napoli, Italy; 4 Dipartimento di Neurologia, Centro Regionale Sclerosi Multipla, Azienda Ospedaliera “A. Cardarelli”, Napoli, Italy; 5 Istituto per l’Endocrinologia e l’Oncologia Sperimentale “G. Salvatore”, IEOS-CNR, Napoli, Italy; 6 Unità di NeuroImmunologia, Fondazione Santa Lucia, Rome, Italy; 7 Institute of Dentistry, Barts and The London School of Medicine and Dentistry, Blizard Institute, Queen Mary University of London, London, UK

**Keywords:** Metabolism, Multiple sclerosis, Blood-brain barrier, Tight junction, Cytoskeleton

## Abstract

**Background:**

Multiple sclerosis (MS) is an autoimmune disease which results from the invasion of the brain by activated immune cells across the endothelial cells (ECs) of the blood-brain barrier (BBB), due to loss of immune self-tolerance. Many reports define the metabolic profile of immune cells in MS, however little is known about the metabolism of the BBB ECs during the disease. We aim to determine whether circulating factors in MS induce metabolic alterations of the BBB ECs compared to a healthy state, which can be linked with disruption of BBB integrity and subsequent immune cell extravasation.

**Methods and results:**

In this report, we used an in vitro model to study the effect of sera from naïve-to-treatment, relapsing-remitting MS (RRMS) patients on the human brain microvascular endothelium, comparing effects to age/sex-matched healthy donor (HD) sera. Our data show that RRMS serum components affect brain endothelial cells by impairing intercellular tightness through the down-modulation of occludin and VE-cadherin, and facilitating immune cell extravasation through upregulation of intercellular adhesion molecules (ICAM-1) and P-glycoprotein (P-gp). At a metabolic level, the treatment of the endothelial cells with RRMS sera reduced their glycolytic activity (measured through the extracellular acidification rate-ECAR) and oxygen consumption rate (oxidative phosphorylation rate-OCR). Such changes were associated with the down-modulation of endothelial glucose transporter 1 (GLUT-1) expression and by altered mitochondrial membrane potential. Higher level of reactive oxygen species released from the endothelial cells treated with RRMS sera indicate a pro-inflammatory status of the cells together with the higher expression of ICAM-1, endothelial cell cytoskeleton perturbation (stress fibres) as well as disruption of the cytoskeleton signal transduction MSK1/2 and β-catenin phosphorylation.

**Conclusions:**

Our data suggest that circulating factors present in RRMS patient serum induce physiological and biochemical alterations to the BBB, namely reducing expression of essential tightness regulators, as well as reduced engagement of glycolysis and alteration of mitochondrial potential. As these last changes have been linked with alterations in nutrient usage and metabolic function in immune cells; we propose that the BBB endothelium of MS patients may similarly undergo metabolic dysregulation, leading to enhanced permeability and increased disease susceptibility.

## Background

Multiple sclerosis (MS) is the most common chronic inflammatory demyelinating disorder of the central nervous system (CNS), mediated by autoimmune T cells reaching the brain parenchyma and attacking the myelin sheath [1]. Key features of the disease include blood-brain barrier (BBB) damage, multifocal inflammation, demyelination, oligodendrocyte loss, reactive gliosis and axonal degeneration [2]. Accumulating evidence suggests a role for metabolic changes in MS; obesity has been proposed as a risk factor for developing MS, through a direct crosstalk between energy metabolism alterations in T lymphocytes and their pro-inflammatory behaviour [3]. Moreover, studies have shown that some hormones, including leptin, insulin and ghrelin, play an important role in the initiation and progression of MS [4]. In this regard, it seems that MS patients present alterations in several components of the glycolytic pathway, including elevated lactate levels [5] in the blood, increased activity of enolase, pyruvate kinase and aldolase in cerebrospinal fluid (CSF) [6] accompanied by the presence of anti-glyceraldehyde-3-phosphate dehydrogenase (GAPDH) autoantibodies in the CSF, leading to downregulation of glycolytic pathways. Moreover, immuno-metabolic analysis on naïve -to -treatment RRMS patients has shown that leptin levels are linked to the severity of disease disability [7]. Migration of activated T regulatory cells (Treg) into inflamed tissue is crucial for their immune-modulatory function [8], this being controlled by distinct metabolic pathways [3]; impaired glycolysis would consequently affect their migration and/or impact on self-recognition. In agreement with such findings, we have recently shown an increased migratory capacity of CD4^+^RORgt^+^ T (T_H_17) cells from RRMS subjects in an in vitro model of BBB [9].

Studies to date have mainly centred on the metabolic status of immune cells, but so far no data have been provided on the metabolic status of the endothelial cells (ECs) of the BBB, the interface between the periphery and the CNS, which shows impaired barrier properties in MS [10]. The BBB is a complex cellular three-dimensional structure composed of ECs, pericytes, astrocytes end-feet and extracellular matrix which separates the brain parenchyma from systemic circulation [11]. Far from being inert, the BBB actively controls the transport of molecules and immune cells from blood to brain parenchyma via the regulation of endothelial junction organization and expression of specific transporters [12, 13]. The extreme tightness of the microcirculation at BBB level is due to the presence of specialized endothelial junctional structures called tight junctions (TJ), a protein complex composed by transmembrane (claudin-5 and occludin) and cytoplasmic (ZO-1, 2 and 3) proteins with cytoskeleton (actin) [11]. In addition, adherens junctions (AJ), composed by VE-cadherin and catenins, also synergise with the TJ in maintaining the barrier [14]. Moreover, ECs are responsible for providing the nutrients necessary for brain function by selectively exchanging molecules with the blood, a task performed mainly by active, energy-consuming transporters. Consequently, not only is there a higher metabolic demand on the ECs of the BBB compared to others in the body, but any failure in the regulation of cerebral endothelial metabolic processes can lead to functional BBB alterations. In this study, we have investigated the impact of circulating factors present in RRMS patients on normal ECs of the BBB. We show that the endothelial cells present altered glycolysis and oxidative respiration, similar to the adaptive cells of the immune system, a change culminating in perturbed barrier function.

## Methods

### Cells

As a general in vitro model of the human BBB, we used hCMEC/D3 cells [15]. hCMEC/D3 were cultured in tissue culture flasks previously coated with 1 μg/ml rat tail collagen type I solution, in EBM-2 complete medium (Lonza). Cultures were maintained at 37 °C in 5% CO_2_, replaced with fresh medium every 3 days, and split when they reached 100% confluence.

### Samples

Blood samples from RRMS patients (adults, newly diagnosed) and healthy donors (HD) were collected following the recommendations of Ethical Committees: Università degli Studi di Napoli “Federico II”. RRMS subjects were enrolled by our clinician collaborator at Universita` degli Studi di Napoli “Federico II”, Azienda Ospedaliera di Rilievo Nazionale “Antonio Cardarelli” Napoli (Italy). Peripheral blood was obtained from RRMS and healthy subjects after they signed a written informed consent approved by the institutional review board, as previously described [9, 16].

The study population consisted of 30 subjects, 62.3% females with an average age of 37.7 ± 12.2 (mean ± s.d.) years and a body mass index (BMI) of 26.4 ± 5.7 (mean ± s.d.) kg/m^2^. All RRMS subjects were at diagnosis and had a Kurtzke Expanded Disability Status Scale (EDSS) [17] score between 0 and 6, with a median of 2.0 and a disease duration of 2.4 ± 4.4 (mean ± s.d.) years. Healthy subjects were matched for age, body mass index (BMI) and sex and had no recent history of inflammation, neither endocrine nor autoimmune disease. The ethnic distribution amongst the groups was comparable with all participants being Caucasian European.

Serum and plasma were isolated from whole blood by density gradient centrifugation and stored at −80 °C. Serum used for all biological tests (transmigration assay, western blotting and ImageStream) was decomplemented (20 min at 56 °C) prior to the experiments. Cells were incubated (16 h) with EBM2 medium containing 20% sera from RRMS patients or matched HD to evaluate the effect of serum components on brain ECs. hCMEC/D3 cells were grown in 6-well or 24-well plates; per well sera were pooled from 2 donors (RRMS or healthy), matching in age, sex and EDSS score. Each well was treated with different pooled sera pairs. *N* numbers in figure legends represent the total number of individual donor sera used. Peripheral blood mononuclear cells (PBMCs) were isolated from whole blood as previously reported [9, 16]. Briefly, blood samples were carefully layered on top of the Ficoll solution (Sigma-Aldrich) and then centrifuged (2000 RPM, 30 min, at room temperature) to form layers containing different cell types. After centrifugation, PBMCs were collected in a whitish layer between the plasma (top) and the erythrocyte (bottom) layers. PBMCs were counted, suspended in FBS solution containing 10% DMSO (Sigma-Aldrich) and stored in liquid nitrogen.

### Transmigration assay

hCMEC/D3 cells were grown (72 h) on Transwell polycarbonate filters (membrane diameter 6.5 mm, 5 μm porosity—Corning, Germany) previously coated with calf skin collagen type I (Sigma-Aldrich) and bovine plasma fibronectin (Sigma-Aldrich). Transmigration assays were run with PBMCs in their autologous sera (AS) or with a swap of sera, i.e., healthy donor PBMC + AS or healthy donor PBMC + RRMS patient sera. EBM-2 complete medium (w/o VEGF) containing 20% sera was added to the lower compartment of Transwell 30 min prior to transmigration assay. Then, PBMCs (1x10^6^ cells) were suspended in EBM2 medium containing sera and added to the upper compartment of Transwell in contact with hCMEC/D3 monolayer. The transmigration assays were run during 4 h at 37 °C at 5% CO_2_. Adhered cells were detached from the top compartment of Transwell by using a 0.2% trypsin solution, whereas transmigrated cells were collected from the bottom compartment by aspirating the medium. Cells were fixed with 2% PFA (10 min at room temperature), and staining was performed for further FACS analysis on the transmigrated cells.

### FACS analysis

hCMEC/D3 cells incubated (16 h) in EBM2 medium containing 20% sera from RRMS patients or matched HD were collected in FACS buffer, fixed with 2% formaldehyde (Sigma-Aldrich) for 10 min at RT and washed with FACS buffer (1% BSA in PBS). ICAM-1 APC (1:100 Bioscience), VCAM-1 Alexa fluor 488 (1:100, eBioscience), Occludin AF 405 (1:100 eBioscience) VeCadherin Alexa fluor 488 (1:100, eBioscience) and P-glycoprotein Alexa fluor 488 (1:100 ebioscience) were added for 30 min at room temperature. Between each step, samples were vortexed and centrifuged for 5 min at 4 °C. Finally, cells were suspended in PBS and analysed by FACS using a LSR-Fortessa (BD Biosciences), with 10,000 events collected on gated cell population.

The expression of markers in PBMCs were measured on fixed cells incubated with staining buffer (PBS + 2% BSA). Cell surface staining for CD4-AF648 was carried out at room temperature for 30 min. After washing, the cells were permeabilised using the human Foxp3 buffer set (BD Biosciences) for 20 min followed by a wash step and staining within intracellular markers Foxp3-PE and RoRγt-BV421. After a final wash, cells were suspended in PBS for FACS analysis. All data was analysed using FlowJo software version 10.

### Seahorse metabolic analysis

The metabolic profile of ECs, from healthy and naïve-to-treatment RRMS subjects, was evaluated through real-time measurements of extracellular acidification rate (ECAR) or oxygen consumption rate (OCR), using an XFe-96 extracellular flux analyzer (Seahorse Bioscience [18]). ECs were plated in XF-96 plates (Seahorse Bioscience) at a density of 2 × 10^4^ cells per well and were cultured in EBM2 medium for 72 h. Sixteen hours prior to the assay, the medium was changed to EBM2 containing 20% sera from RRMS patients or matched HD); individual donor sera were used per well. ECAR was measured in XF medium containing 1 mM glutamine in basal conditions and in response to glucose (10 mM), oligomycin (1 μM) and 2DG (50 mM) (all from Sigma-Aldrich). Metabolic parameters were then calculated from ECAR profile: basal glycolysis post glucose injection (after glucose addition), maximal glycolysis (after oligomycin addition) and glycolytic capacity (calculated as the difference between oligomycin-induced ECAR and 2DG-induced ECAR). OCR was measured in XF medium containing 1 mM sodium pyruvate, 2 mM glutamine and 10 mM glucose in basal conditions and in response to oligomycin (1 μM), FCCP (0.5 μM) and rotenone/antimycin A (0.5 μM each). Metabolic parameters were then calculated from the OCR profile: basal respiration (without addition of any compound), ATP production (calculated as the difference between basal OCR and OCR post oligomycin injection), proton leak (calculated as the difference between OCR post oligomycin injection—OCR post-rotenone/antimycin A injection), maximal respiration (after FCCP addition) and spare capacity (calculated as the difference between FCCP-induced OCR and basal respiration). Experiments with the Seahorse system were done with the following assay conditions: 3 min of mixture; 3 min of waiting; 3 min of measurement.

### Western blot

hCMEC/D3 cells were grown (72 h) on 24-well plates previously coated with calf skin collagen type I and bovine plasma fibronectin. Cells were incubated (16 h) with EBM2 medium containing 20% sera from RRMS patients or matched HD. Following incubation, cells were washed with cold PBS and cells were suspended in RIPA buffer (1% Triton X-100, 1% sodium deoxycholate, 0.1% SDS, 150 mM NaCl, and 50 mM Tris-HCl, and pH 7.2) supplemented with protease inhibitors (aprotinin, leupeptin, PMSF, sodium fluoride and sodium orthovanadate). Cell lysates were centrifuged (900 g at 4 °C for 20 min) and total protein content was determined by the BCA method. Samples (10 μg) were separated by SDS-PAGE (10%), and transferred to a nitrocellulose membrane in a transfer apparatus (Bio-Rad Laboratories, CA, USA). Membranes were incubated (1 h at room temperature) in blocking buffer (TBS-T containing 3% bovine serum albumin), followed by incubation (16 h at 4 °C) with anti-hexokinase-1 (Abcam ab 65069) dilution 1/800, anti-aldolase (Abcam ab169544) dilution 1/2000, anti-enolase-1 (Abcam ab155102) dilution 1/2000, anti-DLAT (Sigma SAB2108113) dilution 1/1000, anti-aconitase-2 (Abcam ab110321) dilution 1/1000, anti-DLST (Abcam ab177934) dilution 1/10000 or anti-ERK1/2 (Cell Signalling Technology #9102) dilution 1/1000. Membranes were developed using ECL^TM^ (GE Amersham) reagent and immunoreactive bands were detected by exposure to photographic film. Band intensities were measured by optical densitometry using the ImageJ (NIH, USA) software, with ERK1/2 being used as the loading control.

### Confocal microscopy

hCMEC/D3 cells were grown on chambered microscope slides coated with 0.1 μg/ml calf skin collagen type I (Sigma-Aldrich) in VEGF-free EBM-2MV medium (Promocell, UK). Cells were stimulated for 24 h with media containing 20% sera from RRMS patients or matching HD (*n* = 3). Cells were fixed in 2% formaldehyde in PBS at 4 °C, rinsed in PBS and incubated with Alexa Fluor 488-conjugated phalloidin (1:200 dilution; Thermofisher Scientific, UK) for 20 min. Nuclei were counterstained with 50 ng/ml DAPI in ddH_2_O and mounted under mowiol. Images were captured using an LSM880 confocal laser scanning microscope (Carl Zeiss Ltd., Cambridge, UK) fitted with 405 and 488 nm lasers and an × 63 oil immersion objective lens (NA, 1.4 mm, working distance, 0.17 mm). Images were captured with the ZEN imaging software (Carl Zeiss Ltd., UK) and analysed using ImageJ 1.51 h (National Institutes of Health, USA).

### Paracellular permeability and TEER

hCMEC/D3 cells were grown on Transwell polycarbonate filters (pore size, 0.4 μm; Sigma-Aldrich) pre-coated with calf skin collagen type I (Sigma-Aldrich) and bovine plasma fibronectin (Sigma-Aldrich). Paracellular permeability of 70 kDa FITC-dextran was assessed after stimulation with 20% sera from RRMS patients or matching HD [19]. Transendothelial resistance across the monolayer was determined using an Endohmeter (World Precision Instruments). Resistance from coated cell-free inserts was always subtracted from the resistance obtained in the presence of endothelial cells.

### Image stream

hCMEC/D3 cells were grown (72 h) on six-well plates previously coated with calf skin collagen type I and bovine plasma fibronectin. Cells were incubated (16 h) with EBM2 medium (LONZA-UK) containing 20% sera from RRMS patients and matched HD. Following incubation, cells were washed three times with cold PBS and detached from plates by using 0.2% trypsin. Cells were collected and incubated (30 min at 4 °C) with staining buffer containing rabbit anti-GLUT-1 antibody (ThermoFisher Scientific, UK). Next, cells were washed with cold PBS, centrifuged, and incubated (30 min at 4 °C) with staining buffer containing Alexa Fluor 488-conjugated anti-rabbit secondary antibody (ThermoFisher Scientific, UK). Cells were fixed with 2% formaldehyde (10 min at room temperature) and permeabilised (20 min at room temperature) with staining buffer containing saponin (2.5 μg/ml). DAPI (4’,6-diamino-2-phenylindole, 50 ng/ml) was used for nuclear staining. Imaging flow cytometry was performed on an ImageStream^x^ Mark II operated by the INSPIRE software (Amnis Corporation). Fluorescence was recorded using excitation with 488 nm laser for AF488/ANXA1 and 405 nm laser for DAPI; emission was collected using a 480-560 nm filter or with a 420-505 nm filter respectively. Bright-field images of cells were collected simultaneously. Samples were run alongside single-stained and unstained control hCMEC/D3 cells, in order to gate different cell populations (negative or positive staining). In each experiment, a template of settings was created and it was applied to all files. A total of 10,000 events were collected for each sample, and data were analysed using the IDEAS Application 6.1 software (Amnis Corporation).

### Nitric oxide measurement

Nitric oxide (NO) production was determined by the Griess reaction, which measures the content of nitrite (NO_2_−), a stable and non-volatile product of NO [20] . Briefly, hCMEC/D3 cells were grown (72 h) on 96-well plates previously coated with calf skin collagen type I and bovine plasma fibronectin. Cells were growth for 48 h with EBM2 medium and further incubated for 16 h with 20% sera from RRMS patients or HD. The supernatant was collected and centrifuged (1000 g at 4 °C). A total of 50 μl/well culture medium was incubated (15 min at room temperature) with Griess reagent (5% phosphoric acid containing 1% sulfanilamide and 0.1% naphthylethylenediamine dihydrochloride). Nitrite levels were determined by comparing the absorbance at 540 nm with a standard curve generated by NaNO_2_; minimum sensitivity was 3 μM nitrite.

### Mitochondria analysis

Mitochondrial mass was assessed by incubating labelled ECs with 100 nM of MitoTracker Green FM (ThermoFisher) for 30 min at 37 °C, 5% CO_2_. Mitochondrial membrane potential was investigated using TMRE (ThermoFisher), 1 μM TMRE was incubated with labelled PBMCs for 30 min at 37 °C, 5% CO_2_. Mitochondrial ROS was measured using MitoSOX Red tracer (ThermoFisher), 2 μM MitoSOX was incubated with labelled hCMEC/D3 cells for 15 min at 37^o^C, 5% CO_2_ in phenol red-free medium, cells were washed with PBS and medium containing 20% serum from healthy controls or RRMS patients was added. MitoSOX fluorescence was monitored every 5 min for a period of 2 h using a CLARIOStar fluorescence plate reader (BMG Labtech, Germany) with excitation and emission wavelengths of 510 nm and 580 nm respectively. Rate of change in fluorescence normalised to individual sample baseline was then compared. Cells treated with 2.5 μM rotenone were used as a positive control.

### Phospho-proteomics

Relative levels of phosphorylation of 43 kinase phosphorylation sites and 2 related total proteins were determined using the human phospho-kinase array kit (R&D System, ARY003B) following the manufacturer instructions. Briefly, hCMEC/D3 cells were grown (72 h) on 6-well plates previously coated with calf skin collagen type I and bovine plasma fibronectin. Cells were incubated (16 h) with EBM2 medium containing 20% sera from RRMS patients and matched HD. Sera were pooled from 10 donors (4 male, 6 female, EDSS score 1.5-2). Then, cells were washed with cold PBS, suspended in lysis buffer and protein content was measured by performing a BCA assay. Nitrocellulose membranes containing spotted capture and control antibodies were incubated (16 h at 4 °C) with cell lysates (400 μg/sample), followed by incubation with a cocktail of biotinylated detection antibodies. Streptavidin-HRP and chemiluminescent detection reagents were applied and the signal of each capture spot was detected by exposure to photographic film. Spot intensities were measured by optical densitometry using the software Image Studio^TM^ Life (LI-COR Biosciences), and the signal of reference spot was used as loading control.

### Statistical analysis

Statistical analyses were performed using GraphPad Prism 8.1 program (La Jolla, CA, USA). Data were analysed using parametric (one-way ANOVA or Student’s *t* test as appropriate) or non-parametric (Mann-Whitney) tests depending on normality distribution (Shapiro-Wilcox). For ANOVA, post hoc analysis was performed using Tukey’s honest significant difference test. Significance was established when *P* value ≤ 0.05

## Results

### RRMS sera treatment induces a pro-inflammatory phenotype in hCMEC/D3 cells impairing the functionality

**Fig. 1:**
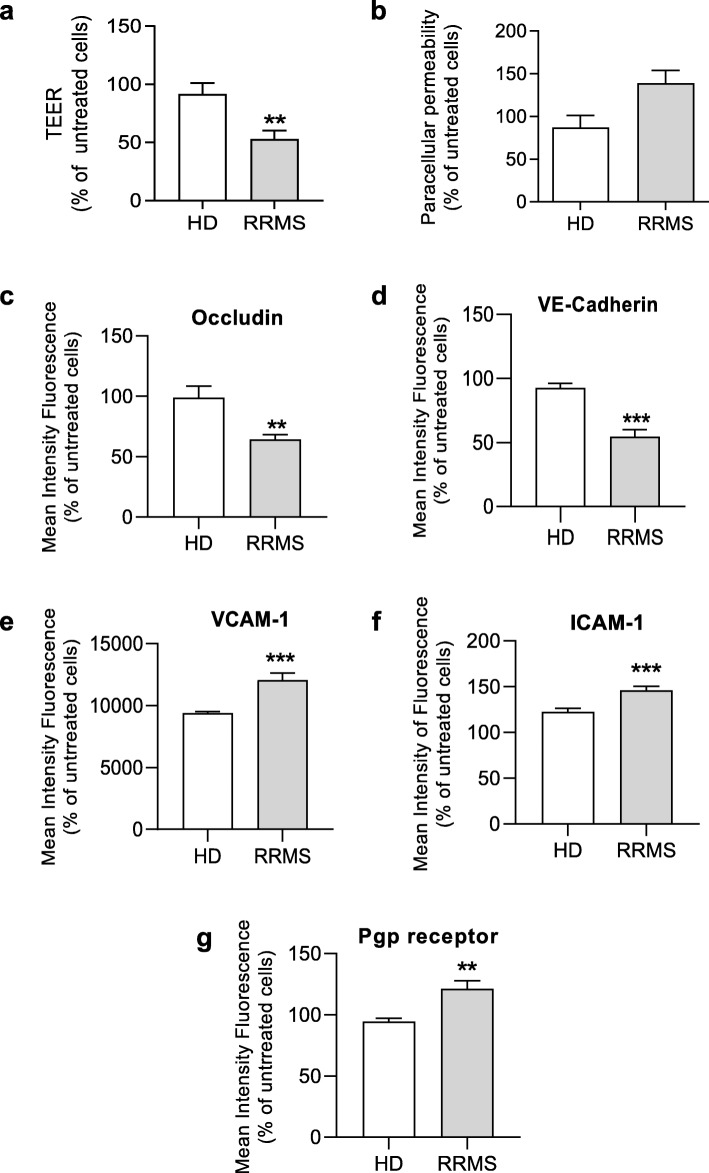
Serum from RRMS patients significantly attenuates BBB function and induces a pro-inflammatory phenotype. Assessment of (**a**) the trans-endothelial electrical resistance (TEER) and (**b**) paracellular permeability of hCMEC/D3 monolayers to 70 kDa FITC-dextran following treatment for 16 h with 20% HD or naïve-to-treatment RRMS sera. **c**-**g** Expression of occludin (**c**), VE-Cadherin (**d**), ICAM-1 (**e**), VCAM-1 (**f**) and P-glycoprotein (Pgp) (**g**) in hCMEC/D3 cells treated with HD or RRMS sera. Data are presented as average of three experiments performed on *n* = 12-24 donors (pooled in pairs matched in sex, age and EDSS score for RRMS patients), expressed as mean + s.e.m. Statistical analysis are performed by Student’s *t* test or by Mann-Whitney *U* test (two-tails)*P ≤ 0.05, **P ≤ 0.01, ***P ≤ 0.001

We have previously reported the presence of circulating immuno-metabolic molecules such as sICAM-1 and leptin in the sera of RRMS patients [7]; in this study, we examined whether such sera would impact the properties of the BBB using an in vitro model. Treatment of hCMEC/D3 immortalised human cerebromicrovascular endothelial cells overnight with 20% sera from naïve-to-treatment RRMS patients (Supplementary Table 1), significantly reduced trans-endothelial electrical resistance (TEER) and increased paracellular permeability to a 70 kDa FITC-dextran tracer (Fig. 1a-b) compared to cells exposed to HD sera. In accordance with these functional increases in permeability, examination of the key intercellular junction components occludin and VE-cadherin revealed that treatment with RRMS sera significantly reduced their expression when compared with cells exposed to HD sera (Fig. 1c-d). Moreover, treatment with RRMS sera significantly enhanced endothelial expression of the adhesion molecules ICAM-1 and VCAM-1 which permit firm adhesion and transmigration of T cells (Fig. 1e-f), indicating a switch towards an inflammatory cell phenotype. A central feature of the BBB is the expression of a wide range of xenobiotic export transporters, of which P-glycoprotein is perhaps the most studied [21]. Expression of this transporter was significantly upregulated by treatment with RRMS sera (Fig. 1g), a change which may also reflect the inflammatory status of the cells as P-glycoprotein is known to facilitate leukocyte migration into the brain parenchyma [21].

**Fig. 2:**
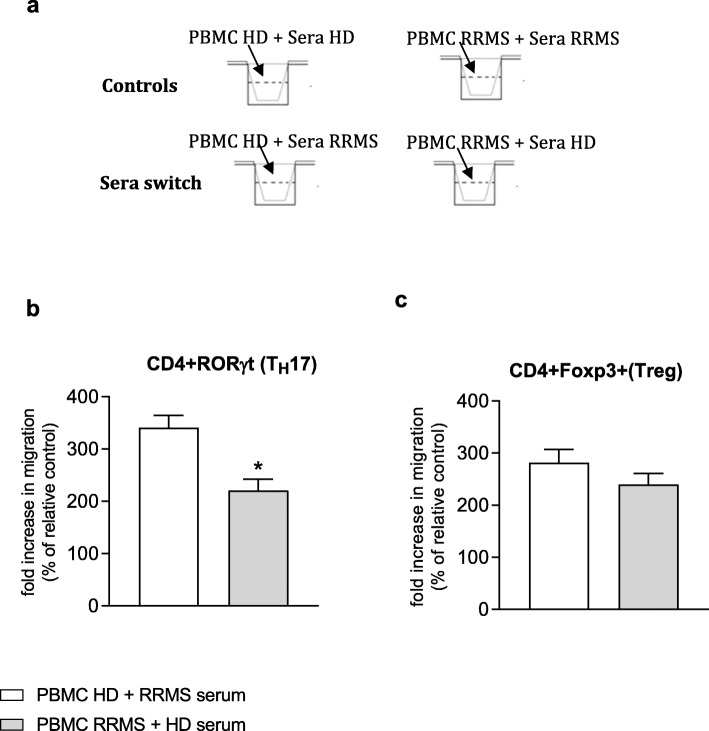
Effect of RRMS sera and matching sex and age HD on PBMCs migration on a normal endothelium. Freshly isolated PBMCs from healthy or RRMS subjects (*n* = 6/group) were put in contact for 4 h with hCMEC/D3 monolayer on Transwell polycarbonate filters in the presence of culture medium supplemented with 20% serum (16 h) from autologous healthy subject (control) or naïve-to-treatment RRMS patient (serum switch effect) (*n* = 6 in duplicate). PBMCs from RRMS patients were placed in contact with autologous RRMS patient serum (control) or healthy subject serum (serum switch effect). **a** Schematic diagram of experiment. After 4 h, the number of (**b**) CD4^+^RoRγt+ (T_H_17) and (D) CD4^+^Foxp3^+^ (Treg) migrated was evaluated by FACS analysis. Data are expressed as fold increase (% of relative control). Statistical analysis are performed by using Mann-Whitney *U* test (two tails) (mean ± s.e.m.); *P ≤ 0.05

As well as regulating passage of solutes and molecules into the brain, the BBB also controls leukocyte migration, a step particularly important for the immune protection of the brain. In MS, such control is lost and we have recently shown that T cells exhibit a pro-migratory profile in RRMS patients [9]. To specifically investigate the dual effects of RRMS sera on both the endothelium and on PBMC, we measured T cell subset migration across hCMEC/D3 cells pre-treated with the sera of RRMS patients or HD. The presence of RRMS patient sera induces healthy subject T helper (T_H_)17 cell migration (CD4^+^ RoRγt^+^) (Fig. 2a) in comparison to similar cells from MS patients incubated in the presence of healthy donor serum. In contrast, the presence of the sera from RRMS patients did not significantly affect the migration of healthy subject T regulatory (Treg) cells (CD4^+^ Foxp3^+^) when compared to RRMS patient cells (Fig. 2b). Such data indicate a role for pro-inflammatory factors present in RRMS sera [7] affecting T_H_17 cells but not Treg cells.

**Fig. 3:**
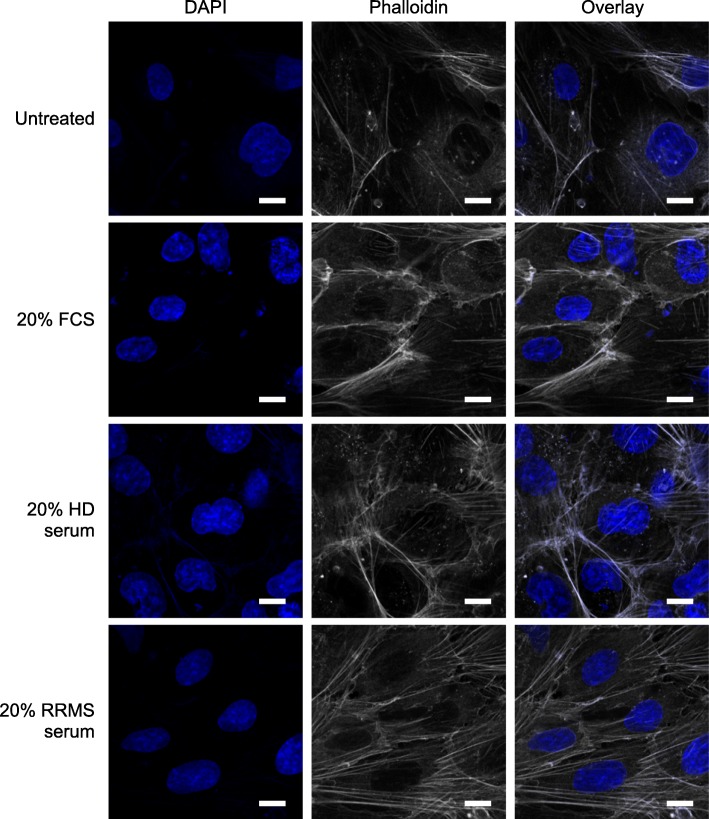
Confocal analysis of F-actin distribution in hCMEC/D3 cells treated with 20% RRMS or HD sera. hCMEC/D3 cells were treated with 20% FCS, 20% HD sera or 20% RRMS sera for 16 h (*n* = 3) and F-actin distribution was defined by AlexaFluor 488-conjugated phalloidin alongside DAPI nuclear counterstain. Scale bar = 10 μm

Central to efficient BBB function is the link between tight junction molecules and the actin cytoskeleton, with the presence of a cortical network of actin fibres being required for efficient junction operation [22]. To explore this aspect, we performed confocal microscopic analysis of F-actin distribution in hCMEC/D3 cells treated with 20% RRMS or HD sera. Notably, exposure to HD sera did not affect the primarily cortical arrangement of F-actin fibres seen in untreated cells, but treatment with RRMS sera showed a clear alteration in F-actin distribution, with a marked increase in cytosolic stress fibre appearance (Fig. 3), suggesting a level of stress upon the cells.

### RRMS sera modifies brain endothelial cell metabolic profile

**Fig. 4:**
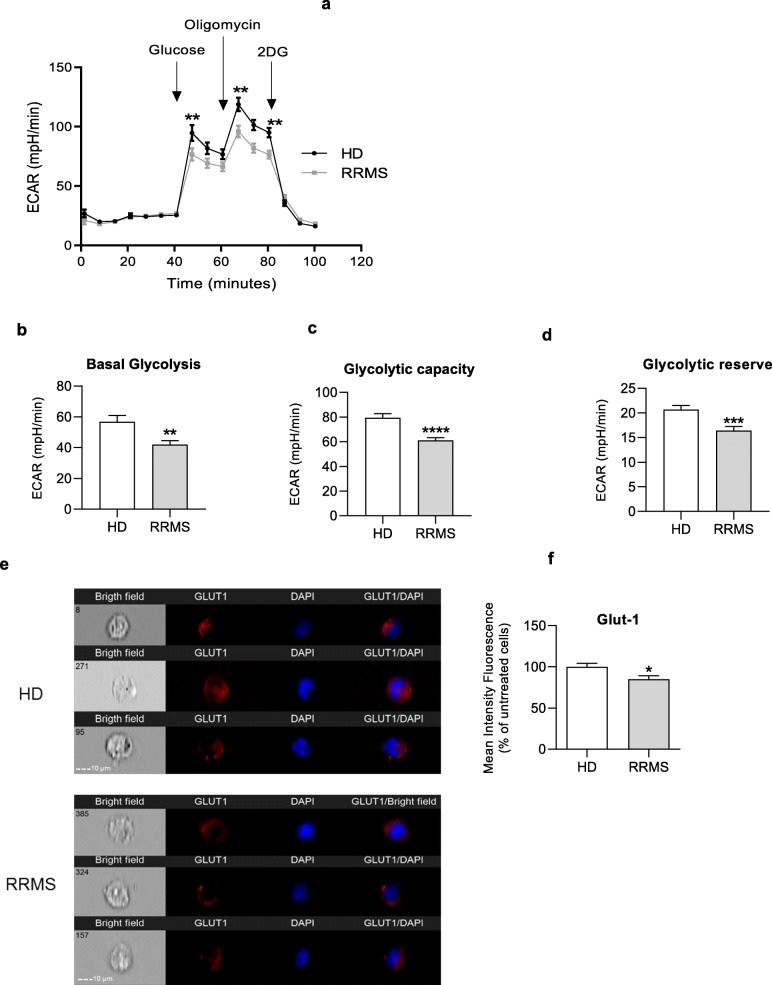
RRMS sera induce impaired engagement of glycolysis in ECs from the BBB. **a** Kinetic profile of ECAR in hCMEC/D3 cells treated overnight with 20% HD or RRMS naïve-to-treatment sera. Real time ECAR was measured, under basal conditions and in response to glucose, oligomycin and 2-DG. Indication of glycolytic pathway activation, calculated from hCMEC/D3 ECAR profile: (**b**) basal glycolysis, (**c**) glycolytic capacity and (**d**) glycolytic reserve. Data are presented as average of three experiments performed on 25 donors/group, matched in sex, age and EDSS score. Data are shown as mean ± s.e.m. Statistical analysis was performed by paired Wilcoxon test **P ≤ 0.01, ***P ≤ 0.001). **e**, **f** Sera isolated from RRMS patients downregulate GLUT1 expression on membrane of brain ECs. hCMEC/D3 monolayer plated on 24-well plate were treated (16 h) with sera isolated from RRMS patients and matched HD. Cells were collected and stained with rabbit anti-GLUT-1 antibody. DAPI was used for nuclear staining. **e** Imaging flow cytometry was performed on an ImageStreamx Mark II operated by INSPIRE software (Amnis Corporation). **f** Data represent % of mean intensity of fluorescence (MIF) in comparison to sera from matched HD. Statistical analysis was performed using Mann-Whitney *U* test (two-tails) *P < 0.05 vs HD, *n* = 5/group

Having identified clear signs that brain endothelial cells exposed to RRMS sera are under stress, we examined possible explanations for these effects. Key to cellular resilience is an efficient energy supply, and both glycolysis and mitochondrial activity in BBB endothelial cells are critical for their functional activity [23]. Moreover, impairments in cellular energy reserve/spare capacity have been related to a range of pathologies affecting high energetic requirement tissues such as the brain [24]. We and others have shown T cells of RRMS patients exhibit lower glycolytic rates than those of healthy subjects [3, 6, 9]; hence, we evaluated whether RRMS sera had a similar effect on ECs through studying glycolysis via measurement of the extracellular acidification rate (ECAR) and oxidative respiration measured via oxygen consumption rate (OCR). Treatment with RRMS sera caused lower glycolytic activity compared to treatment with HD sera (Fig. 4a-d), data which correlated with lower expression of the glucose uptake transporter GLUT-1 in RRMS sera treated hCMEC/D3 cells than those treated with HD sera (Fig. 4e-f).

**Fig. 5:**
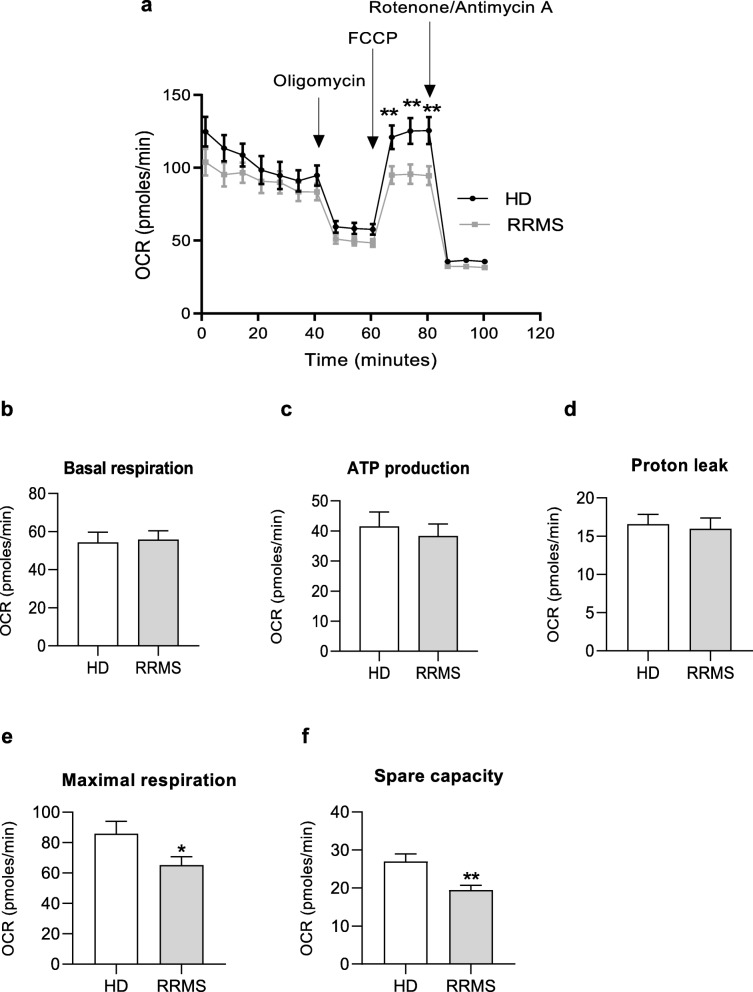
RRMS sera induce an altered mitochondrial respiration in ECs. **a** Kinetic profile of OCR in hCMEC/D3 cells treated overnight with 20% HD or RRMS naïve-to-treatment sera. Real time OCR was measured, under basal conditions and in response to oligomycin, FCCP, Antimycin A and Rotenone. Indices of mitochondrial respiratory function, calculated from hCMEC/D3 OCR profile: basal and maximal respiration (**b**, **e**), ATP production (**c**), proton leak (**d**) and spare capacity (**f**). Graphics are a cumulative data performed on 10-15 donors/group, matched in sex, age and EDSS score. Data are shown as mean ± s.e.m. (statistical analysis by Mann-Whitney *U* test (two-tails)*P ≤ 0.05, **P ≤ 0.01)

Having identified changes in glycolytic rate induced by exposure to RRMS sera, we examined whether the other principal cellular energy pathway, mitochondrial oxidative respiration, would be similarly affected. Compared with hCMEC/D3 cells treated with 20% HD sera, RRMS sera significantly reduced maximal oxygen consumption (Fig. 5e) revealing a loss in spare capacity (Fig. 5f), an estimate of the potential bioenergetic reserve the cell can call upon, without affecting ATP production rates or proton leakage (Fig. 5b-d).

### RRMS sera impairs brain endothelial cell mitochondrial function

**Fig. 6:**
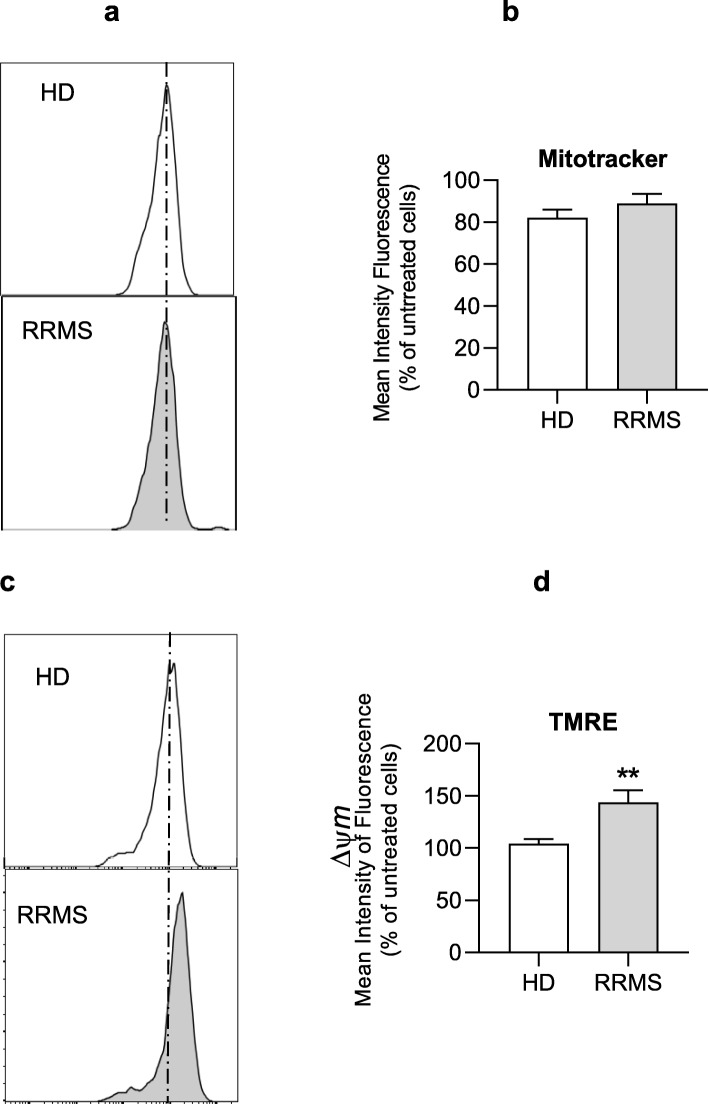
Impact of RRMS sera treatment on the mitochondria content and membrane potential in brain ECs. (**a**, **b**) Representative flow cytometry plots and cumulative graphs of mitotracker green staining in hCMEC/D3 cells treated with 20% RRMS sera or HD for 16 h. (**c**, **d**) Representative flow cytometry plots and cumulative graphs of TMRE staining showing membrane potential in hCMEC/D3 treated with RRMS or HD sera. Data are expressed as mean ± s.e.m. of 30 donors/group (pooled in pairs matched in sex, age and EDSS score for RRMS patients). Statistical analysis by Mann-Whitney *U* test (two-tails) **P ≤ 0.01

The mitochondria are the main site of oxygen consumption in a cell; hence, we examined their structural and functional integrity in response to RRMS sera. Using MitoTracker Green, a mitochondrial-specific dye that binds to mitochondrial membrane independently of mitochondrial membrane potential, we found no difference in mitochondrial mass between ECs treated with RRMS or HD sera (Fig. 6a and the histogram).

**Fig. 7:**
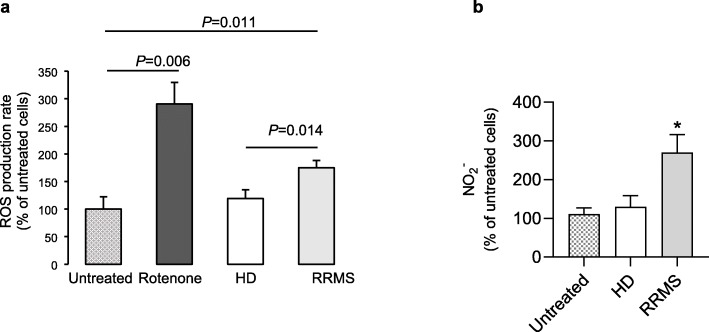
RRMS sera stimulate ROS production in ECs of BBB. **a** ECs were preloaded with 2 μM Mitosox red, then incubated for 2 h at 37 °C, fluorescence measured every 5 min and rate of fluorescence increase determined. ECs were either untreated (EBM-2MV medium only), stimulated with 2.5 μM rotenone (mitochondrial complex I inhibitor; positive control), or treated with 20% human serum in EBM-2MV medium. Data are mean ± s.e.m., *n* = 7 for untreated and rotenone, *n* = 10 for HC or RRMS samples. Data analysed by one-way ANOVA with Tukey’s HSD post hoc test. *p* = 0.014 RRMS vs HD, *p* = 0.011 vs untreated. **b** NO production in hCMEC/D3 cells treated overnight with 20% serum from RRMS naïve-to-treatment or HD, determined by Griess assay. Data are expressed as mean + s.e.m. of duplicates, one representative from three independent experiments (*n* = 5-8 donors/group), (Mann-Whitney *U* test (two-tails)*P ≤ 0.05

As the mitochondrial membrane potential (Ψm) generated by proton pumps (complexes I, III and IV) is essential for energy mobilisation during oxidative phosphorylation [20], we investigated how brain endothelial cell Ψm was affected by incubation with RRMS sera using the tracer TMRE. ECs exposed to 20% RRMS sera had a significantly higher proportion of hyperpolarised mitochondria than cells treated with HD sera (Fig. 6c-d). Hyperpolarised mitochondria can be a source of reactive oxygen species (ROS), which can potentially cause cellular damage [25]; hence, we examined the impact of RRMS sera exposure upon hCMEC/D3 cells. Mitochondrial superoxide production, measured using MitoSOX Red, was found to be significantly higher in ECs treated with RRMS sera, whilst no differences were found between untreated and HD sera treated ECs (Fig. 7a). Similarly, production of nitric oxide (NO-measured through analysis of its stable metabolite, nitrite) was significantly greater in ECs treated with RRMS sera than those exposed to HD sera (Fig. 7b).

### Signalling behind the effect of RRMS sera on ECs change

**Fig. 8:**
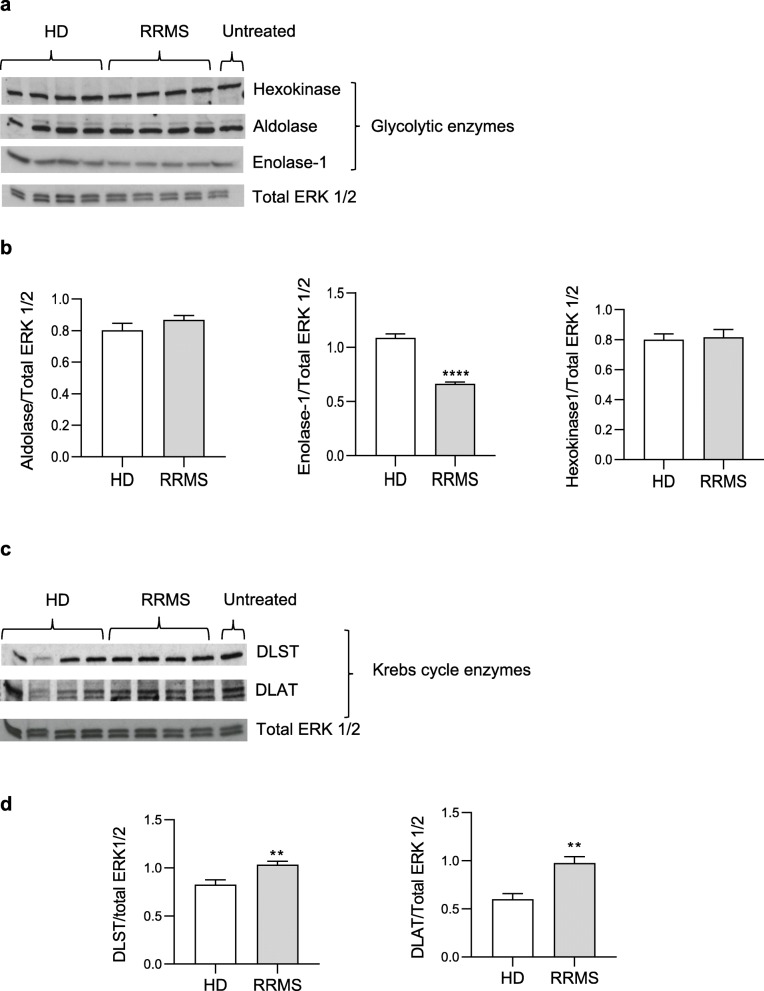
Biochemical pathways of brain ECs treated with RRMS sera and matched HD. **a** Immunoblot for *h*exokinase, *a*ldolase and *e*nolase-1 on hCMEC/D3 cells treated overnight with 20% HD or RRMS naïve-to-treatment sera. Total ERK 1/2 served as a loading control. One representative out of at least three independent experiments performed on 12 donors in total/group. **b** Densitometry quantification for *h*exokinase, *a*ldolase and *e*nolase-1 performed on all 10 donors in total/group. **c** Immunoblot for DLST and DLAT, on hCMEC/D3 cells treated overnight with 20% HD or RRMS naïve-to-treatment sera. Total ERK 1/2 served as a loading control. One representative out of at least three independent experiments performed on 12 donors in total/group. **d** Densitometry quantification of DLST and DLAT normalized on total ERK 1/2 performed on all 10 donors. Data are expressed as mean ± s.e.m. (*s*tatistical analysis by Mann-Whitney U test (two-tails) *P ≤ 0.05)

To interrogate the alterations in glycolysis (ECAR) and oxidative respiration (OCR) described above, we investigated expression of key glycolytic and Kreb’s cycle enzymes in hCMEC/D3 cells treated for 16 h with 20% RRMS or HD sera. Analysis of glycolytic enzymes revealed a marked and specific reduction in enolase-1 expression upon RRMS sera exposure, an effect not seen for hexokinase or aldolase (Fig. 8a, b), accompanied by significant increases in expression of the Kreb’s cycle enzymes DLST and DALT (Fig. 8c, d).

Microvascular integrity is dependent on tight and adherens junction assembly which is closely related with a correct cytoskeleton organization [26]. Additionally, there is extensive evidence for the interaction of metabolic enzymes with eukaryotic cytoskeleton [27].

**Fig. 9:**
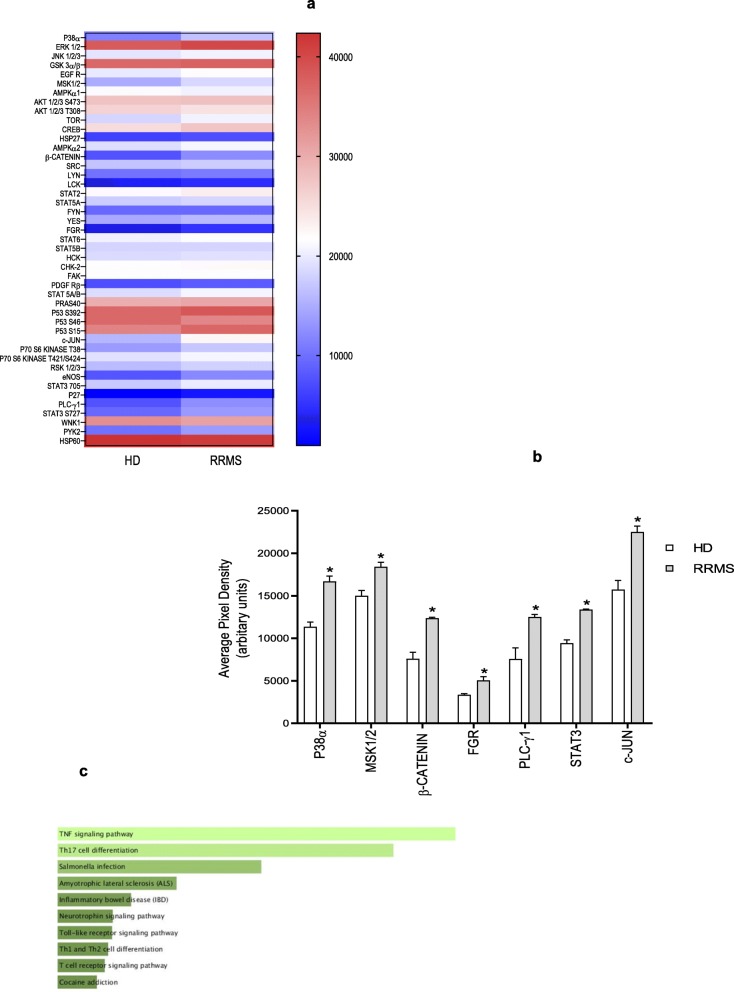
Phospho-protein activation. **a** The map display the expression of differentially expressed proteins identified from the proteomics analysis in hCMEC/D3 cells treated overnight with 20% RRMS naïve-to-treatment or HD sera performed on 10 donors pooled per group pooled (in sex, age and EDSS score for RRMS patients), in duplicate. The colour scale illustrates the expression level of each protein across the 2 samples; the colour intensity indicates the degree of protein up- or downregulation. **b** Average pixel density of phosphorylated proteins known to be involved in pro-inflammatory processes/pathways significantly different. **c** Association of all significantly phosphorylated enzymes with KEGG pathways, ranked in order of p value, Enrichr. Data are shown as mean ± s.e.m. (statistical analysis by Mann-Whitney *U* test (two tails) *P ≤ 0.05, **P ≤ 0.01

To examine the possible signalling cascade coordinating disrupted metabolism, cytoskeletal remodelling and endothelial cell junction assembly, we performed phospho-proteomic analysis of hCMEC/D3 cells treated with either 20% RRMS or HD sera. Activation of a number of signalling molecules was detected (Fig. 9a), amongst which the most significant were p38α, MSK1/2, β-catenin, FGR, PLCγ1, STAT3 and c-JUN (Fig. 9a, b). Notably amongst these, the tyrosine kinase FGR has been shown to regulate cellular fuel choice, permitting cells to respond to energy depletion [28] and β-catenin is well known to be required for maintaining adult blood-brain barrier integrity and CNS homeostasis [28]. Using Enrichr [29, 30] to examine KEGG pathways associated with the list of proteins phosphorylated after RRMS serum exposure, we identified significant over-representation of several inflammation-related pathways (Fig. 9c), supporting our previous identification of a pro-inflammatory EC phenotype.

Together, these data support our hypothesis that factors present in the serum of RRMS patients can exert a detrimental impact upon brain EC physiology, reducing their energy reserves and placing them in a condition of metabolic stress, effects which contribute to a deterioration in their key physiological role, provision of an effective permeability barrier. These changes correlate with a clear appearance of a pro-inflammatory phenotype, and facilitate immune cell binding and extravasation, directly contributing to the progression of MS disease.

## Discussion

The pathogenesis of MS is still unknown but several cellular mechanisms have been proposed, including genetic factors, viral infections, autoimmune attack, demyelination, mitochondrial dysfunction, free radical production, ionic imbalance and cellular clearance system dysfunction [31–33]. While all these contribute to the demyelinating and neurodegenerative characteristics of the disease [34], one of the established trademarks is the presence of profound abnormalities in the cerebral ECs [35]. These ultimately result in the alteration of normal BBB function, permitting transendothelial migration of activated leukocytes into the CNS, where they drive inflammation and ultimately lead to neurodegeneration [36].

Accumulating evidence suggests that changes in the metabolism of immune cells, such as normal metabolic pathway alteration and loss of key metabolites, contributes to the pathogenesis of autoimmunity, resulting in a loss of immune tolerance to self [36]. Indeed, it was recently demonstrated that T cells from naïve-to-treatment RRMS patients displayed impaired engagement of glycolysis and mitochondrial metabolism upon T cell receptor (TCR) activation, associated with an impaired generation of Treg cells and a consequent loss of immune self-tolerance [3, 6]. Furthermore, the presence of higher levels of pro-inflammatory factors, such as leptin, sCD40L, MCP-1, IL-6 and sTNF-R [7] in RRMS sera and the specific link with metabolic alterations in the immune system of MS patients suggests MS can be considered a chronic metabolic disorder [37].

The aim of the current work was to verify whether circulating factors found in MS patients [7] affect BBB structure, function and metabolism. Using the established hCMEC/D3 in vitro model of the human BBB [38], we show for the first time, to our knowledge, that RRMS serum factors significantly impair BBB integrity, promote a pro-inflammatory cellular phenotype, and markedly weaken the ability of the endothelial cells to maintain an adequate energy supply.

Brain microvascular ECs, being the first contact with the circulation, possess highly organized tight and adherens junctions, in order to maintain the restrictive properties of the BBB. In MS, the presence of pro-inflammatory factors, activated immunocompetent cells and reactive nitrogen species result in modifications to the integrity and organization of these junctional molecules (occludin and claudin and Ve-Cadherin) and consequently enhanced BBB permeability [15]. We show here that the sera from naïve-to-treatment RRMS patients induced alterations to BBB permeability and regulated essential mediators of the tightness of the BBB (occludin and VE-cadherin). These effects were most probably mediated by the presence of circulating factors in RRMS serum, known to include pro-inflammatory cytokines such as IL-6 and CD40L, as described previousl y[7]. Moreover, this milieu upregulates adhesion molecules, such as ICAM-1, favouring the firm adhesion of leukocytes and their transmigration into the brain parenchyma. Another molecule involved in T cell trafficking is P-gp, an efflux pump that actively removes toxic compounds from the brain at the cost of ATP hydrolysis [21], which has also been shown to regulate CD4+ and CD8+ T cell migration into the brain through release of CCL2 [21]. In agreement with this, we have shown an increase in P-glycoprotein expression upon treatment with RRMS serum. Together, the data we present in this study strongly indicate the induction of a pro-inflammatory endothelial phenotype by RRMS serum, emphasising the presence of pro-inflammatory factors in the circulation of MS patients.

Interestingly, the functional ECAR/OCR results seen in ECs post-RRMS serum exposure resemble the immuno-metabolic profiles of T cells from RRMS patients [6], namely presenting impaired glycolysis and reduced expression of the transporter GLUT-1. These effects may be caused by the pro-inflammatory molecules present in the sera of patients affected by MS [5]. At the mitochondrial level, hCMEC/D3 cells treated with RRMS sera displayed a reduction in maximal respiration and spare respiratory capacity, further suggesting the inability of the cells to respond to an energetic demand. Nonetheless, while at a biochemical level, we found an increase in Kreb’s cycle enzyme expression; this did not translate into changes in ATP production. This may reflect an attempt by the cells to try to compensate for the impaired glycolytic function by enhancing Kreb’s cycle activity, or synthesizing other intermediates for cytokines.

Our data indicate that RRMS serum factors place endothelial cells under energetic stress, with a marked reduction in glycolytic reserve and mitochondrial spare capacity, accompanied by a notable loss of mitochondrial membrane potential. This strongly indicates that the cells have less resilience to challenge, such as inflammation. Importantly, depletion of reserve respiratory capacity through mitochondrial dysfunction has been shown to underlie increased BBB permeability in animal models of occlusive stroke and endotoxaemia [39]. This idea is strongly supported by our findings that whilst mitochondrial mass was unchanged by RRMS sera exposure, clear signs of mitochondrial dysfunction are present, including mitochondrial membrane hyperpolarisation and a loss in basal and maximal respiration rate and respiratory spare capacity. Similarly, the increase in mitochondrial membrane potential identified by increased TMRE accumulation also indicates a degree of mitochondrial stress, as suggested by increased superoxide and nitric oxide production, leading to activation of signaling pathways driving changes in energy metabolism.

The changes we have seen in the endothelial cells may have consequences for the other members of the neurovascular unit. We have studied an in vitro model of endothelial cells in isolation, whereas in the true BBB, the function of these cells is supported and regulated by the other cell types, especially pericytes and astrocytes [38]. Production of high levels of ROS and nitric oxide by endothelial cells operating under conditions of metabolic stress may well be harmful for these other cell types, further weakening BBB properties.

## Conclusions

**Fig. 10:**
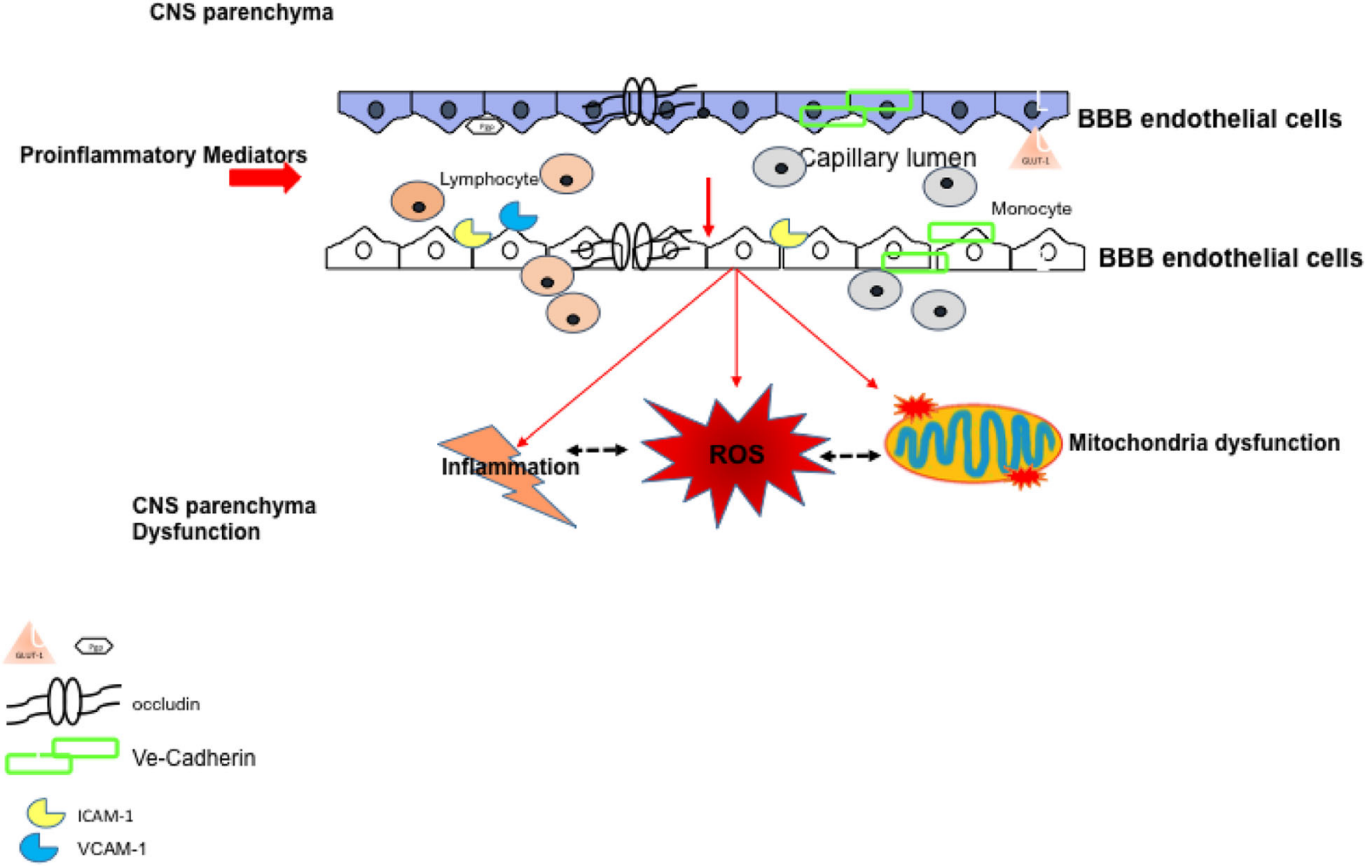
Schematic diagram. Schematic representation of potential-triggered signals in RRMS patients responsible for the alteration in function and biochemistry of the ECs of the BBB. Pro-inflammatory mediators circulating in RRMS patients will affect the essential components of the BBB ECs named occludin, VE-cadherin as well as P-glycoprotein (P-gp). Adhesion molecules are also upregulated facilitating the adhesion and transmigration trough disruption of tight and adherens junctions. RRMS sera factor (pro-inflammatory and endocrine such as leptin) will also impact on the ECs metabolism down modulating the GLUT-1 receptor and triggering an impaired glucose (ECAR) and oxidative respiration (OCR) damaging the mitochondria and prompting the release of reactive species (ROS) which will further disrupt the neurovascular unit (basal lamina and astrocytes end-feet) targeting the neurons in the brain parenchyma

Our data show that the endothelial cells of the BBB exposed to circulating factors present in the sera of RRMS patients undergo a perturbation of mitochondrial function and oxidative respiration, associated with cytoskeletal dysfunction, enhanced barrier permeability and induction of a pro-inflammatory phenotype. We propose (see scheme Fig. 10) that in MS the BBB disruption may be secondary to circulating immune factors which are primary to the chain of events contributing to the disease. Dampening inflammation and controlling endocrine factors may help repair the BBB damage with a beneficial effect on disease control and progression.

## Supplementary information

**Additional file 1: Supplemental Table 1.** Characteristic of naïve to treatment RRMS and healthy subjects.

## Data Availability

Data sharing is not applicable to this article as no datasets were generated or analysed during the current study.

## Abbreviations

BBB: Blood-brain barrier
M: Multiple sclerosis
RR: Relapsing-remitting
HD: Healthy donors
ICAM-1: Intracellular adhesion molecule 1
VCAM-1: Vascular cell adhesion molecule 1
TEER: Trans-endothelial resistance
PBMC: Peripheral blood mononuclear cells
EC/hCMEC-D3: Human brain endothelial cells
ECAR: Extracellular acidification rate
OCR: Oxygen consumption rate
P-gp: Pglycoprotein
GLUT-1: Glucose transporter-1
MSK1/2: Mitogen stress-activated kinases 1 and 2
Treg: Regulatory T cells
T conv: Conventional T cells
TH17: T helper 17 cells
DLST: Dihydrolipoamide succinyltransferase
DLAT: Dihydrolipoamide S-acetyltransferase
