# Supplementary material for: Immuno-metabolic impact of the multiple sclerosis patients’ sera on endothelial cells of the blood-brain barrier
Source: J Neuroinflammation. 2020 May 9;17:153. doi: 10.1186/s12974-020-01810-8 (PMC7210692; doi:10.1186/s12974-020-01810-8)
Supplement: Supplementary file 1 — Additional file 1: Supplemental Table 1. Characteristic of naïve to treatment RRMS and healthy subjects. [file 12974_2020_1810_MOESM1_ESM.docx]

|  | RRMS subjects (n=30) | Healthy Subjects (n=30) |
| --- | --- | --- |
| Male | 9 | 9 |
| Female | 21 | 21 |
| BMI Mean (kg/m^2^)  Mean ± s.d. | 25.0 ± 4.5 | 24.8 ± 3.1 |
| EDSS at baseline  Median [min; max] | 2 [0.0-5.0] | - |
| Disease duration,  mean (year) | 2 | - |

**Supplemental Table 1**.Characteristic of naïve to treatment RRMS and healthy subjects
